# The Epidemiology of Major Depressive Episode in the Iraqi General Population

**DOI:** 10.1371/journal.pone.0131937

**Published:** 2015-07-31

**Authors:** Ali Obaid Al-Hamzawi, Ronny Bruffaerts, Evelyn J. Bromet, Abdulzahra Mohammed AlKhafaji, Ronald C. Kessler

**Affiliations:** 1 Al-Qadisia University, College of Medicine, Diwania, Iraq; 2 Universitair Psychiatrisch Centrum—KULeuven (UPC-KUL), Leuven, Belgiumbstract; 3 Department of Psychiatry, State University of New York at Stony Brook, Stony Brook, NY, United States of America; 4 Department of Health Care Policy, Harvard Medical School, Boston, MA, United States of America; University of South Florida, UNITED STATES

## Abstract

**Objective:**

To assess the prevalence, symptom severity, functional impairment, and treatment of major depressive episode (MDE) in the Iraqi general population.

**Methods:**

The Iraq Mental Health Survey is a nationally representative face-to-face survey of 4,332 non-institutionalized adults aged 18+ interviewed in 2006–2007 as part of the WHO World Mental Health Surveys. Prevalence and correlates of DSM-IV MDE were determined with the WHO Composite International Diagnostic Interview (CIDI).

**Findings:**

Lifetime and 12-month prevalence of MDE were 7.4% and 4.0%, respectively. Close to half (46%) of the 12-month MDE cases were severe/very severe. MDE was more common among women and those previously married. Median age of onset was 25.2. Only one-seventh of 12-month MDE cases received treatment despite being associated with very substantial role impairment (on average 70 days out of role in the past year).

**Conclusions:**

MDE is a commonly occurring disorder in the Iraqi general population and is associated with considerable disability and low treatment. Efforts are needed to decrease the barriers to treatment and to educate general medical providers in Iraq about the recognition and treatment of depression.

## Introduction

In the past decades, there has been a rapid demographic and epidemiological transition in the Arab world that encompasses a significant increase of non-communicable diseases, with mental disorders–and especially depressive disorders–as conditions that merit attention from both policy planners and clinicians [1,2]. Globally, depression is ranked the 11^th^ leading cause of Disability Adjusted Life Years worldwide, but, interestingly, the 3th in the Middle East region [3]. Despite the fact that depression is less extensively studied in the Arab and the Middle Eastern countries, there are indications that the disorder is quite common in the region. General population studies have been carried out in quite a number of Arab and Middle Eastern countries [4–14]. Based on these studies, median 12-month and lifetime estimates of depression (both Major Depressive Disorder [MDD] and Major Depression Episode [MDE]) are 4.7 and 8.9%, respectively. Depressive disorders were more prevalent among females (with a ratio of 4:1), those with lower education, or those never married. Especially persons with war experiences were at higher risks [9,14].

Virtually no data exist on the prevalence of depression in Iraq, except one lifetime estimate of MDD, i.e. [15]. Rudimental knowledge on comorbidity, burden, treatment, or associated suicidal behaviors of depression is non-existing. Nonetheless, such data are useful to study the impact of war on the general population. Since the early nineties of the previous century, and especially between 2003 and 2010, the war in Iraq has had a dramatic impact on the country resulting in decreased health status of the Iraqi people but also in the destruction of many of the country’s (mental) healthcare system [2]. In line with this, basic but essential vital health data can guide governments to proactive healthcare planning in further elaboration the strategic plan by the Iraqi Ministry of Health.

In the light of these limitations, the current study examines the epidemiology of MDE in the general population of Iraq. We use data from the Iraq Mental Health Survey (IMSH) that was conducted in 2006–2007 as part of the World Health Organization (WHO) World Mental Health (WMH) Survey Initiative (www.hcp.med.harvard.edu/WMH). The WMH comprises a series of psychiatric epidemiologic studies conducted in countries throughout the world. This study builds on an earlier study on mental disorders in Iraq [15] in which basic descriptive data on prevalence and correlates of mental disorders were investigated. The present study examines: (a) the 12-month and lifetime prevalence of MDE in the Iraqi general population; (b) socio-demographic risk factors; (c) comorbid mental disorders and suicidal behaviors, (c) associated impairments in role functioning, and (d) patterns of treatment.

## Materials and Methods

### Sampling and procedures

The Iraqi Mental Health Survey (IMHS; 18+ years, n = 4,332) was carried out in 2006–2007 by the Iraq Ministry of Health, the Iraq Central Organization for Statistics and Information Technology (COSIT), the Ministry of Health of the Kurdistan region (MoHK), the Kurdistan Regional Statistics Office (KRSO), and the College of Medicine at Al-Qadisiya University in conjunction with the WHO World Mental Health Surveys. Procedures for obtaining oral informed consent and protecting individuals were approved and monitored for compliance by the Institutional Review Boards of the organizations coordinating the survey. Permission for the study was granted by the Institutional Review Boards (IRB) of the Iraq Ministry of Health, the Iraq Central Organization for Statistics and Information Technology (COSIT), the Ministry of Health of the Kurdistan region (MoHK), the Kurdistan Regional Statistics Office (KRSO), and the College of Medicine at Al-Qadisiya University. Standardized descriptions of the goals and procedures of the study, data uses and protection, and the rights of respondents were provided in both written and verbal form to all predesignated respondents before obtaining verbal informed consent for participation in the survey. Oral consent was documented by the trained lay-interviewer. The survey was administered to a stratified multistage clustered area probability sample of household residents in the central and southern governorates in August-September 2006, in Anbar in October-November 2006, and in the Kurdistan region during February-March 2007. The response rate was 95.2%. More details about sampling are provided elsewhere [16].

All interviews were administered face-to-face by trained lay interviewers using training and field quality control procedures described in previous reports [17–19]. Training of local trainers and supervisors took place at the WMH Data Coordination Center at the University of Michigan (USA).

### Measurements

#### Mental disorders

The WHO Composite International Diagnostic Interview Version 3.0 (CIDI) was the instrument administered in the survey [17]. The CIDI is a fully structured diagnostic interview that assesses mental disorders, their treatment, and a wide range of possible risk factors. The WHO translation, back-translation, and harmonization protocol was used to translate instruments and training materials. One of the implications of this approach procedure is that those characteristics that described the core symptoms of MDE were customized when the original wording did not match local language use. However, we did not alter any of the DSM-IV MDE criteria [20].

The disorders analyzed in this study are DSM-IV mood disorders (MDE and dysthymia), anxiety disorders (generalized anxiety disorder [GAD], panic disorder and/or agoraphobia, posttraumatic stress disorder [PTSD], and social phobia), alcohol abuse and/or dependence, and externalizing disorders (attention deficit disorder and intermittent explosive disorder) [21]. Clinical reappraisal studies carried out in four WMH countries provided evidence showing good concordance between CIDI-3.0 diagnoses and diagnoses based on blinded re-interviews, with area under the receiver operator characteristics curve ranging between 0.73–0.93 for lifetime mood/anxiety disorders, and 0.83–0.88 for 12-month mood/anxiety disorders [22].

#### Suicidality

Suicidality was assessed using the CIDI 3.0 suicidality module [17]. This module includes an assessment of the 12-month occurrence of serious suicide ideation, plans, and attempts. Respondents who endorsed a 12-month history of suicidal ideation were classified as suicidal.

#### Role impairment

Role impairment was assessed by two measures. First, the WHO disability assessment schedule (WHO-DAS-2) to assess functional impairments in 5 domains during the past 30 days [23]. The first domain includes the number of days in the past 30 days when the respondent was partially or completely unable to work or carry out their normal activities because of physical or mental health problems. The remainder includes severity-persistence estimates of impairments in self-care (e.g. bathing, dressing), mobility (e.g. standing, walking), cognition (e.g. concentrating, remembering), and social functioning (e.g. conversing, maintaining emotional control while around others). The 5 WHO-DAS-2 scales were transformed to a theoretical range of 0 (no impairment at any time in the past 30 days) to 1.0 (complete inability to perform the functions throughout the full 30 days). Second, Respondents with MDE in the past 12 months were also administered the Sheehan Disability Scale (SDS) [24] to evaluate the degree of impairment in functioning due to depression in four domains during the worst month of the past year: work, household, close relationships, and social roles. Responses were scored as mild (1−3), moderate (4−6), severe (7−9) and very severe (10).

#### Symptom severity

Respondents with 12-month MDE were administered a modified version of the Quick Inventory of Depressive Symptomatology Self-Report (QIDS-SR) to assess symptom severity in the worst month of the past year [25]. The QIDS-SR is a fully-structured measure that is strongly related both to the clinician-administered Inventory of Depressive Symptomology (IDS-C) [26] and to the Hamilton Rating Scale of Depression (HRSD) [27]. Transformation rules developed for the QIDS-SR [28] were used to convert scores into clinical severity categories mapped to conventional HRSD ranges (i.e. mild, moderate, severe/very severe).

#### 12-month treatment

Respondents were asked about 12-month treatment for emotional problems. The number and duration of 12-month visits were also assessed. Responses were used to classify 12-month treatment in the specialty mental health sector (inpatient treatment or outpatient treatment with a psychiatrist, psychologist, any other mental health professional, or a social worker or counselor in a mental health specialty setting, or use of a hotline), the general medical sector (outpatient treatment with a primary care physician, other medical specialist, nurse, or any other health professional not previously mentioned), human services sector (outpatient treatment with a religious or spiritual advisor or with a social worker or counselor in any setting other than a specialty mental health setting), and the complementary-alternative medical (CAM) sector (outpatient treatment with any other type of healer, participation in an internet support group, or participation in a self-help group).

### Statistical methods

Cross-tabulations were used to calculate prevalence, comorbidity, symptom severity, impairment, and treatment. The Kaplan-Meier method was used to generate age-at-onset curves. We used logistic regression analysis to study socio-demographic correlates of prevalence and treatment. The regression coefficients were transformed to odds ratios (ORs) for ease of interpretation. Confidence intervals (Cis) were estimated using the Taylor series linearization method implemented in the SUDAAN software package [29]. Multivariate significance tests were calculated using Wald chi^2^ tests based on coefficient variance-covariance matrices that were adjusted for design effects using the Taylor series method. Since the IMHS was carried out using a multi-stage cluster design, and every individual was assigned a known non-zero probability of selection, all data were analyzed using the design weights for the survey. Standard errors were estimated using the Taylor series linearization method to adjust for design effects [30]. All statistical analyses were carried out in STATA version 9.2. Statistical significance was based on 2-sided design-based tests evaluated at the .05 level of significance.

## Results

### Description of the sample

The sample consisted of 4,332 respondents with an average age of 36.9 (range of 18–96), with equal numbers of men and women and 65.6% married. About eight in ten (i.e. 78.2%) had at least 12 years of education. Whereas 68.2% of men were employed, most women were not (86.9%). The survey did not assess religion given the sensitive nature of the topic at the time of data collection. More details on sample characteristics can be found elsewhere [15,31].

### Prevalence of MDE and sociodemographic risk factors

Prevalence estimates were 7.4% for lifetime and 4.0% for 12-month MDE (Table 1). Almost 6 in 10 lifetime cases (or 57.4%) experienced an episode of MDE in the prior 12-month period. We found a linear increase of lifetime prevalence with age. Prevalence was higher in women than in men (OR = 1.7 [1.0–2.8]). Respondents who were previously married (i.e. separated, widowed, or divorced) were three times as likely to have 12-month MDE (OR = 3.0 [1.6–5.6]) as compared to married respondents. We did not find other sociodemographic correlates.

**Table 1:** 12-month and lifetime prevalence of DSM-IV major depressive episode (MDE) in the Iraqi general population, by cohort.

	12-month MDE (1)			Lifetime MDE (1)		12-month MDE among lifetime MDE	
Cohort	Denominator (1)	n	%(SE)	n	%(SE)	n	%(SE)
18–34	2148	70	2.8 (0.5)	126	4.9 (0.6)	70	57.6 (7.5)
35–49	1332	69	5.3 (0.9)	115	8.4 (1.0)	69	63.6 (6.9)
50–64	589	27	5.3 (1.3)	66	12.0 (2.1)	27	43.9 (6.8)
65+	263	21	6.5 (1.5)	34	13.0 (2.1)	21	49.8 (8.8)
All ages	4332	187	4.0 (0.4)	341	7.4 (0.6)	187	54.8 (4.2)
significance			Chi^2^ = 2.6, p = .064		Chi^2^ = 9.7, p < .001		Chi^2^ = 2.3, p = .088

(1) MDE refers to Major Depressive Episode

### Age-of-onset of MDE and cohort effects

**Fig 1:**
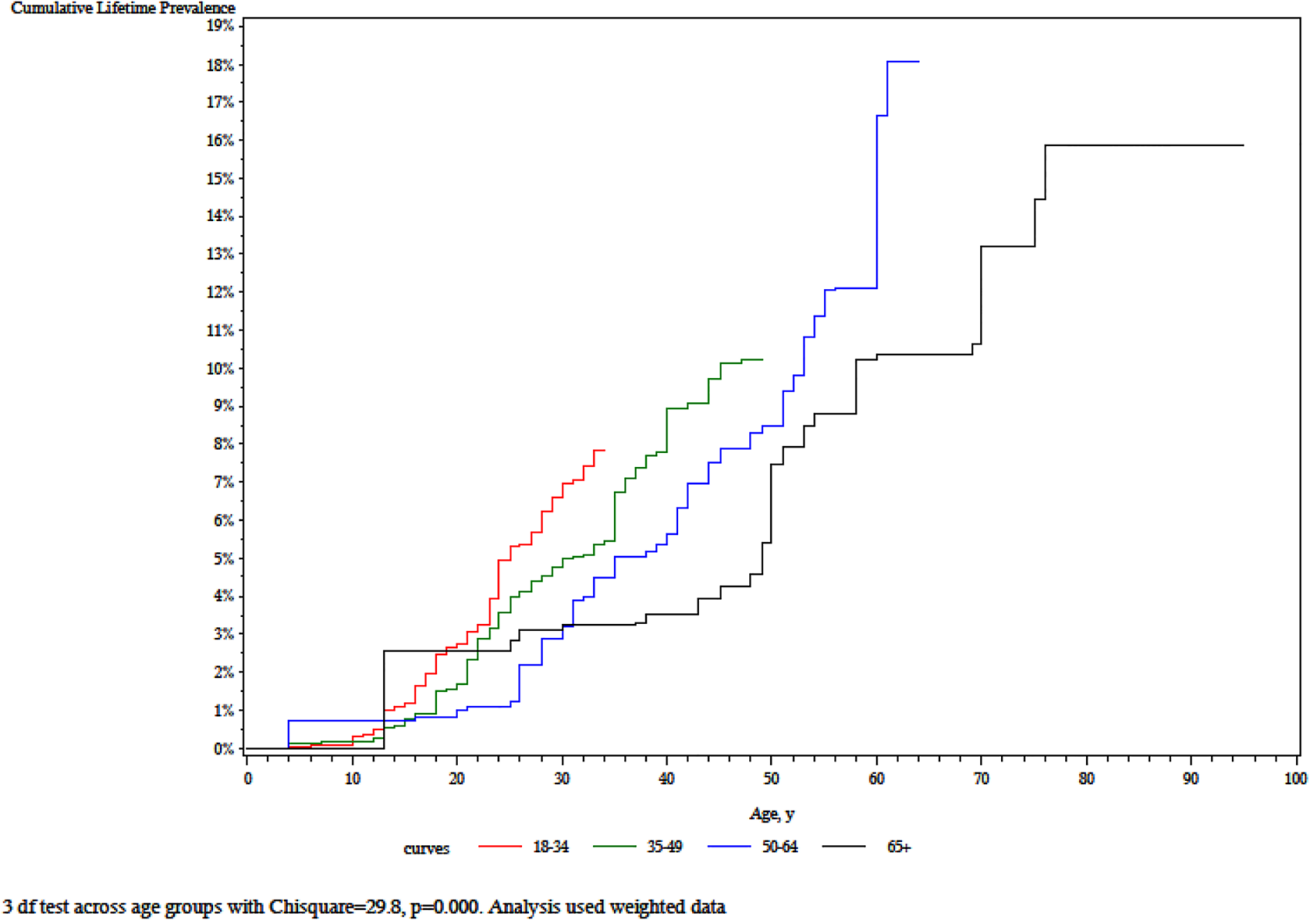
Cumulative lifetime prevalence of DSM-IV major depressive by birth cohort in Iraq.

Mean age of onset was 29.8 years (SE = 1.1) and the mean duration of an episode was 22 weeks (SE = 2.4). The cumulative lifetime prevalence curves are significantly different from each other (χ^2^ = 29.8, p < .001), with in general low risks before the teen years, and then increasing steep slopes in the younger cohorts (Fig 1). We also found a monotonic increase in lifetime prevalence with cohort (i.e. 18–29, 30–44, 45–59, and 50+), from 4.9% in the youngest up to 13.0% in the oldest group (Table 1).

### Psychiatric comorbidity and suicidality

Approximately 6 in 10 respondents with 12-month MDE also met criteria for another 12-month mental disorder, with anxiety disorders, and especially GAD, as the most common comorbid disorder (i.e. 31.1% of those with MDE also meet criteria for GAD, OR = 24.8 [14.2–43.5]) (Table 2). Significant comorbidity associations were also found between MDE and substance abuse and impulse control disorders but we should be careful in interpreting these because of the very low numbers in each cell. Only 23 respondents (or 7.3% among those with MDE; OR = 8.3[4.4–15.8]) also met criteria for PTSD. Further, among those with past year MDE, 7.6% reported suicidal ideation in the same timespan (OR = 11.9 [4.9–29.0]), 2.2% reported suicide plan, (OR = 14.5 [5.4–39.1]), and 2.6% suicide attempt (OR = 16.7 [4.8–58.1]).

**Table 2:** Comorbidity patterns of DSM-IV major depressive episode with other DSM-IV mental disorders and suicidal behaviors in the Iraqi general population.

12-month disorders	12-month MDE (1)	# with comorbid disorder	% (SE)	OR (95%CI)	significance
Dysthymia	187	16	7.5 (3.4)	0.0 (0.0–0.0)	Chi^2^ = 84.2, p < .001
Any mood disorder	187	20	11.2 (2.5)	0.0 (0.0–0.0)	Chi^2^ = 88.1, p < .001
Generalized anxiety disorder	187	58	31.1 (5.1)	24.8 (14.2–43.5)	Chi^2^ = 131.7, p < .001
Specific phobia	187	30	17.4 (5.0)	6.7 (3.2–14.0)	Chi^2^ = 26.7, p < .001
Social phobia	187	6	5.1 (2.5)	12.7 (3.9–41.7)	Chi^2^ = 18.4, p < .001
Post-traumatic stress disorder	187	23	7.3 (1.8)	8.3 (4.4–15.8)	Chi^2^ = 44.1, p < .001
Panic disorder	187	14	11.0 (4.1)	20.5 (8.8–48.0)	Chi^2^ = 50.7, p < .001
Any anxiety disorder	187	99	50.7 (6.1)	16.1 (9.4–27.5)	Chi^2^ = 107.0, p < .001
Any substance disorder	187	2	2.5 (2.4)	15.3 (1.8–128.3)	Chi^2^ = 6.6, p = .010
Intermittent explosive disorder	187	9	4.7 (2.4)	3.4 (1.0–11.1)	Chi^2^ = 4.1, p = .043
Any impulse disorder	187	10	5.1 (2.4)	3.6 (1.2–11.3)	Chi^2^ = 5.2, p = .022
Any disorder	187	111	58.5 (5.8)	18.4 (11.1–30.5)	Chi^2^ = 134.5, p < .001
Exactly 1 disorder	187	68	34.9 (6.8)	12.9 (6.8–24.7)	Chi^2^ = 62.8, p < .001
Exactly 2 disorders	187	32	14.6 (3.9)	14.5 (5.4–39.1)	Chi^2^ = 102.9, p < .001
3+ disorders	187	11	9.0 (3.9)	16.7 (4.8–58.1)	Chi^2^ = 48.0, p < .001
12-month suicidal behaviors					
Suicidal ideation	187	24	7.6 (2.8)	11.9 (4.9–29.0)	Chi^2^ = 31.0, p < .001
Suicidal plans	187	10	2.2 (0.9)	14.5 (5.4–39.1)	Chi^2^ = 29.2, p < .001
Suicidal attempt	187	8	2.6 (1.2)	16.7 (4.8–58.1)	Chi^2^ = 20.4, p < .001

(1) Specific disorders with n<5 are omitted in Table 2. Any mood disorder also includes bipolar disorder (n = 5), any anxiety disorder also includes agoraphobia without panic (n = 4), any substance disorder also includes alcohol abuse disorder (n = 1) and drug abuse disorder (n = 1), any impulse disorder also includes attention deficit disorder (n = 2).

### Role impairment and severity

Analyses on the WHO-DAS-2 components (Table 3) showed that recent MDE (i.e. meeting MDE criteria in the 30 days before the interview) is associated with statistically significant impairments in all 5 WHO-DAS-2 domains (compared with respondents who never met criteria for MDE). Respondents with recent MDE had scores that were on average 4 times higher than the WHO-DAS-2 scores of those who never met MDE criteria. Additional analyses revealed that the average number full out of role days in respondents with MDE in the year before the interview was 70.0, with severe or very severe cases being associated with most days out of role (83.2 days per year) compared to mild (20.5 days per year), and moderate cases (79.0 days per year). In addition, the proportion of respondents with severe or very severe impairment in at least one of the role domains on the SDS was one in four (i.e. 25.9%) among those with mild QIDS-SR scores, about one in two (i.e. 53.3%) among those with moderate QIDS-SR scores, and the vast majority (i.e. 85.8%) among those with severe or very severe QIDS-SR scores.

**Table 3:** Thirty-day standardized comparisons of functional impairment by the WHO-DAS-2 among respondents with vs. without DSM-IV major depressive episode in the Iraqi general population.

		Past 30 days		Past 12 months		>12 months ago		No lifetime MDE	
WHO-DAS-2 domains	n	Mean(SE) and 95% CI	n	Mean(SE) and 95% CI	n	Mean(SE) and 95% CI	n	Mean(SE) and 95% CI	F_3,55_ across 4 recency categories	Significance
Out of role	83	44.2 (SE = 7.7) (1)	104	42.2 (SE = 8.0) (1)	154	23.4 (SE = 3.4) (1)	3991	12.3 (SE = 0.6)	25.6	p < .001
Self-care	83	8.5 (SE = 3.0) (1)	104	16.7 (SE = 5.8) (1)	154	4.1 (SE = 2.2)	3991	1.0 (SE = 0.1)	3.9	p = .013
Mobility	83	18.8 (SE = 4.3) (1)	104	16.7 (SE = 5.1) (1)	154	10.8 (SE = 1.6) (1)	3991	3.0 (SE = 0.2)	12.4	p < .001
Cognition	83	9.7 (SE = 2.2) (1)	104	7.5 (SE = 2.7) (1)	154	4.2 (SE = 1.1) (1)	3991	1.0 (SE = 0.1)	16.5	p < .001
Social functioning	83	8.6 (SE = 2.5) (1)	104	10.1 (SE = 2.9) (1)	154	3.9 (SE = 1.7) (1)	3991	0.6 (SE = 0.1)	6.7	p < .001
Total WHO-DAS-2 score	83	18.0 (SE = 2.8) (1)	104	18.6 (SE = 4.0) (1)	154	9.3 (SE = 1.1) (1)	3991	3.6 (SE = 0.2)	26	p < .001

Abbreviations: DSM-IV: Diagnostic and Statistical Manual of Mental Disorders, Fourth edition; MDE = Major Depressive Episode; WHO-DAS-2, World Health Organization-Disability Assessment Schedule-2

(1) Significantly different from respondents with no lifetime MDE at the .05 level, 2-sided test

As summarized in Table 4, about 86% of the respondents with 12-month MDE were independently classified by the QIDS-SR as clinically depressed during the worst month of the year, with 16.1% mild, 23.7% moderate, and 45.9% severe or very severe. Compared to the mild and moderate cases, severe or very severe MDE was associated with more days out of role (up to 83.2 days per year), a higher role impairment (up to 85.8% with severe or very severe impairment on at least one SDS role domain), and more comorbidity, but did not last longer than average (i.e. 22 weeks).

**Table 4:** Distributions and correlates of symptom severity of 12-month DSM-IV major depressive episode in the Iraqi general population.

	Mild MDE	Moderate MDE	Severe or very severe MDE	Total	
	Mean (SE)	Mean (SE)	Mean (SE)	Mean (SE)	Significance across severity
QIDS distribution of severity (1)	16.1 (4.4)	23.7 (6.2)	45.9 (5.4)	100.0(0.0)	—-
Duration (weeks) (2)	20.6 (6.4)	17.4 (4.9)	24.4 (2.6)	22.0 (2.4)	F = 2.3, p = .113
WHO-DAS-2 Days full out of role (3)	20.5 (10.2)	79.0 (43.1)	83.2 (25.5)	70.0 (17.6)	F = 4.9, p = .011
SDS percentage of role impairment (4)	25.9 (9.4)	53.3 (11.0)	85.8 (5.7)	67.8 (5.6)	chi^2^ = 25.2, p < .001

(1) Percent of people with the Quick Inventory of Depressive Symptomatology- Self Report (QIDS-SR) symptom severity domain.

(2)Number of weeks depressed in the 365 days before the interview.

(3)Number of WHO-DAS-2 days fully out of role (i.e. unable to work or carry on usual activities because of MDE) in the 365 days prior to the interview

(4)Percent who reported severe or very severe impairment in at least one Sheehan Disability Scales (SDS) role domains.

### 12-month treatment

Only 25 out of the 187 MDE cases reported to have received services in the past year. Given this limitation in statistical power, close to one in seven (or 14.5%) of the MDE cases received treatment in the past year (Table 5), with more severe cases receiving treatment than non-severe cases (19.4 versus 7.7%, respectively). MDE cases were mostly treated in specialized mental health settings (8.6%) and human services settings (4.7%). General medical and CAM settings were less frequently used providers.

**Table 5:** 12-month treatment patterns for DSM-IV major depressive episode in the Iraqi general population.

Treatment sectors	# respondents with MDE	# respondents with MDE in treatment	% (SE)	OR (CI) (1)	Significance
Specialty sector	187	8	8.6 (4.1)	27.3 (7.4–101.1)	Chi^2^ = 25.6, p < .001
General medical sector	187	7	3.7 (2.4)	6.0 (1.3–27.2)	Chi^2^ = 5.6, p = .018
Human services sector	187	12	4.7 (2.3)	5.9 (1.7–20.4)	Chi^2^ = 8.2, p = .004
CAM sector	187	1	0.1 (0.1)	1.9 (0.2–24.1)	Chi^2^ = 0.3, p = .600
Any treatment in any sector	187	25	14.5 (4.7)	9.2 (3.9–21.8)	Chi^2^ = 26.5, p < .001

(1) The OR refers to the odds of being treated in one of the indicated sectors for respondents with 12-month Major Depressive Episode compared to those without the disorder.

## Discussion

This is the first study providing information about the epidemiology of MDE in Iraq based on a nationally representative sample using a standardized methodology. We found that MDE is a common mental disorder in Iraq, annually affecting around 475,000 Iraqi adults, of which 46% are severe or very severe cases. The disorder generally starts around the age of 25 (with remarkably higher risks for those born in more recent cohorts) with higher risks for those previously married and female persons.

In general, our data on prevalence of MDE are consistent with the earlier studies in Arab and Middle East countries [4–14]. We also found consistency with previous literature that women and those previously married were at higher risk for MDE. The OR between MDE and being previously married is somewhat higher in our study than the one commonly reported; this may be related to the societal position and associated social difficulties of divorced people, especially in women in a patriarchal society [32,33]. Our data are also in line with studies performed globally, with MDE emerging as a highly impairing disorder with a considerable comorbidity [21,34–37]. In terms of the proportion of 12-month cases among lifetime cases, the IMHS data provide roughly comparable estimates than those commonly found (i.e. 57% in the IMHS and 40–55% in previous studies [36,38,39], suggesting that MDE is a episodically chronic recurrent disorder for the majority of persons. We also confirm the relatively young age at which MDE generally starts, although MDE in Iraq seems to start 5 years earlier than in Lebanon [12]. Comparable data of other countries are not available. Data on the impairments associated with MDE are in line with other evidence that this disorder is burdensome. We did find however that Iraqi respondents with MDE reported more than twofold days out of role per year compared to what is generally reported [35–37].

There are some results that are not consistent with earlier reported data. First, available research shows that being exposed to war is associated with up to fourfold higher rates of MDE [9,10,40–42]. Against estimations that armed violence in Iraq resulted in nearly 350,000 deaths, disabled persons, and injuries between 2003 and 2010 [2,43], we would have expected a higher rate of MDE cases in our survey than we actually did. We do not know why exactly this is the case but one explanation could be that we underestimated the prevalence of MDE because of the bereavement criterion stipulated in DSM-IV [44]. Given that bereavement is an exclusion criterion of MDE, it may be that a number of respondents may have been excluded from MDE because their episode lasted under 2 months and was classified as bereavement instead of MDE [45]. Another possibility is that stigma-related concerns, i.e. not disclosing emotional problems to others, may lead to a significant underreporting of MDE [46]. To the extent that this is the case, the prevalence of MDE might be higher than the one we actually estimated. As this is the first study that aims to estimate the prevalence of MDE in the general population of Iraq, further studies should confirm or refute our findings. Second, we found a rather atypical comorbidity pattern of MDE as only a few respondents met criteria for both MDE and substance abuse disorder. This could either be a real difference or it might be an artifact because of a possible systematic under-reporting related to the non-acceptability of use of alcohol and substances [47]. The other factor for the low prevalence could be the protective role of cultural and religious practices that preclude use of these substances. In any case, this finding remains for further research to clarify. In addition, only 7% of the MDE cases also met criteria for PTSD. The scarce data available from other studies using general populations with [15] but also without war experiences [48] suggest stronger associations between MDE and PTSD than those we found. A third discrepancy with literature is that the associations between MDE and suicidal behaviors appeared to be stronger in this study (i.e. in the 11.9–16.9 range) than in other general population studies in developing countries (i.e. in the 1.5–3.2 range) [49]. Although we should be careful in interpreting this because of the low numbers, this finding confirms the idea that suicidal behaviors and MDE are strongly associated in (post)war times [50]. Taken together, although prevalence estimates are comparable to earlier reports, our findings suggest that MDE in Iraq is characterized by a few specific features, i.e. starting at earlier age age than commonly reported, stronger associated with days out of role than average, and stronger associated with suicidal behaviors than commonly found. To what extent these features are war-related is unknown and speculative, but they support the hypothesis that impairments of MDE may be exacerbated in people with war experiences. This should be investigated in future studies.

One of the most important findings from this study is the dramatically low treatment rate for MDE. Only 1 in 7 MDE cases received any form of mental health care, even if we include treatment received by CAM providers. Comparable figures have been reported from other countries in the region (e.g. Lebanon [51]) but, nonetheless, this low treatment rate is a matter of great concern. The low level of receipt of care may be due to several factors, including lack of awareness about the medical nature of depression [52], stigma associated with the receipt of care [53], or the lack of acknowledging any need for treatment [54]. Still, a prominent reason for the unmet need for care is likely to be the difficulty in accessing relevant services. Moreover, the limited availability of general health services and in particular mental health services, especially in small cities and towns where there are no psychiatrists and psychiatric facilities, is likely to limit the use of the services [55]. Remarkably, male and female respondents had similar probabilities of receiving treatment, a finding that contradicts the common relationship between female gender and the use of services [56]. Cultural issues factors may explain why women may be more reluctant to seek professional help, especially for mental health reasons. Women were found to be more likely to seek help from traditional healers than men [33] but it may also be that disclosing negative emotions is more likely to be accepted within the family than with third parties. Although we have not yet investigated specific reasons for not seeking help, our findings so far strongly suggest that the unmet need for MDE may be considerable, and that there might be significant inequalities to detect in further study. In a hospitalized-based healthcare system, health reforms in Iraq face a call for realignment of the entire health system [2], with a great emphasis on access to care. Acknowledging that it may not be easy to increase the proportion of depressive people seeking professional help in a country that has suffered from wars and economic sanctions, different strategies–maybe implemented in tandem with increased treatment resources and the national health plan–will need to be implemented to target barriers that prevent people from receiving available care [57].

Our study should be interpreted within the context of the following three main limitations. First, mental disorders were assessed using structured interviews by trained lay interviewers. Although previous CIDI validation studies have shown acceptable validity and reliability [23,58], there may be an underestimation of lifetime prevalence because of the effect that self-reported data on mental health or quality of life data may be flawed [59]. Second, our results may be biased because the survey was administered in the two official languages of Iraq (Arabic and Kurdish) whereas Assyrian Neo-Aramaic and South Azeri are recognized regional languages, aside from different other regional languages spoken by Iraqi citizens. Furthermore, individuals with mental disorders could have more frequently rejected to participate in the survey. Although weighting strategies were used to optimize the representativity of the general population, it is plausible that our results were biased because persons with a history of mental disorders might have been less likely to participate [60,61]. A third limitation pertains to the assessment of MDE. It might have been that the use of stem questions in the screening section of the CIDI-3.0 have led to underestimates of MDE [21]. The interview translation, back-translation, and harmonization process in the WMH surveys included customization of the wording used to describe the core MD symptoms based on clinical experiences of local collaborators and the results of pilot studies [19]. However, we made no attempts to develop cut-off points in the CIDI diagnostic algorithms for different countries or to go beyond the DSM-IV criteria to develop distinct criteria for different countries that might have increased our ability to detect depression or depression-equivalents. Last, since people with depression may experience cognitive impairment and somatic symptoms as core symptoms, we might have excluded sub-threshold or atypical cases of depression that would otherwise have qualified for being a case and treatment in a clinical setting. Although clinical reappraisal studies were carried out, no such study was performed in the Iraqi survey.

## Conclusions

MDE is a common disorder in the Iraqi general population, associated with significant impairments in daily life. We found an especially strong association between MDE and suicidal behaviors but this finding needs to be studied in more detail further on. Despite this major burden of MDE, still a very low proportion actually receive treatment. There are good reasons to believe that access to care may be amongst the most important barriers that prevent people from effectively seeking help. One major implication then may be the emphasis on the need for sustainable and structural efforts aimed at increasing access to care, not just for people with MDE, but probably also for other (severe) mental disorders and suicidal behaviors.
